# Effects of Cereal, Fruit and Vegetable Fibers on Human Fecal Weight and Transit Time: A Comprehensive Review of Intervention Trials

**DOI:** 10.3390/nu8030130

**Published:** 2016-03-02

**Authors:** Jan de Vries, Anne Birkett, Toine Hulshof, Kristin Verbeke, Kernon Gibes

**Affiliations:** 1 De Vries Nutrition Solutions, Inc., 7213 CE Grossel, The Netherlands; 2 Kellogg Company, Battle Creek, MI 49015, USA; anne.birkett@kellogg.com (A.B.); toine.hulshof@kellogg.com (T.H.); kernon.gibes@kellogg.com (K.G.); 3 Department of Clinical and Experimental Medicine, 3000 Leuven, Belgium; kristin.verbeke@med.kuleuven.be

**Keywords:** dietary fibers, regularity, bowel function, cereal fiber, vegetable fiber, fruit fiber, fermentation, transit time, fecal weight

## Abstract

Cereal fibers are known to increase fecal weight and speed transit time, but far less data are available on the effects of fruits and vegetable fibers on regularity. This study provides a comprehensive review of the impact of these three fiber sources on regularity in healthy humans. We identified English-language intervention studies on dietary fibers and regularity and performed weighted linear regression analyses for fecal weight and transit time. Cereal and vegetable fiber groups had comparable effects on fecal weight; both contributed to it more than fruit fibers. Less fermentable fibers increased fecal weight to a greater degree than more fermentable fibers. Dietary fiber did not change transit time in those with an initial time of <48 h. In those with an initial transit time ≥48 h, transit time was reduced by approximately 30 min per gram of cereal, fruit or vegetable fibers, regardless of fermentability. Cereal fibers have been studied more than any other kind in relation to regularity. This is the first comprehensive review comparing the effects of the three major food sources of fiber on bowel function and regularity since 1993.

## 1. Introduction

Dietary fiber has well-known beneficial effects on human regularity, increasing fecal weight and transit time [1,2]. Both fecal weight and transit time are key indicators of intestinal and digestive health [3]. Abnormalities serve as diagnostic criteria for prevalent gastrointestinal disorders, including functional constipation [4,5], as well as irritable bowel syndrome and dyspepsia [6].

Dietary fiber plays an important role in the adequate function of the gastrointestinal tract [7] and has been advocated for improved bowel function since the early 1970s [8]. That it improves nutrition and health is without dispute [9]. This review includes fibers that fit the generally agreed upon definition: carbohydrates that are not hydrolyzed or absorbed in the upper part of the gastrointestinal tract [10]. As heterogeneous groups of compounds with unique and overlapping functions, dietary fibers optimize health and provide the best outcomes when they complement and augment each other [9].

Nutrition guidelines from the United States [11] and Europe [12] call for consumers to meet their daily dietary fiber intake goals by eating a variety of fruits, vegetables and whole grains. Cereal fibers and bran are known to increase fecal weight and speed transit time [2,13], but the impact of fruits and vegetables on regularity is largely unknown.

Here, we systematically reviewed the literature and conducted weighted linear regression analyses to assess the effects of dietary fiber from cereals, fruits and vegetables on fecal weight and transit time in healthy individuals. To our knowledge, this is the most comprehensive investigation of interventions on markers of regularity and the intake of dietary fiber from common food sources. The last such review was published over 20 years ago [1].

## 2. Methods

### 2.1. Search Strategy

We split our literature search into two parts: one on cereal dietary fibers; the other on all other fibers. We included studies on all dietary fibers and bowel function published up to the end of 2013, with no lower limit on the publication date. We eliminated duplicates and articles with overlapping results. Reference lists were checked for relevant reports using the snowball method. Two independent reviewers screened titles and abstracts for relevance. We conducted searches according to the PRISMA (Preferred Reporting Items for Systematic Reviews and Meta-Analyses) guidelines [14].

### 2.2. Data Extraction

Publications that met predefined criteria were entered into the FileMaker Pro Advanced 13.0 v5 database (FileMaker, Inc., Santa Clara, CA, USA) and exported to Excel files for further analysis. Relevant data extracted from each publication included: first author; hypothesis; gender; sample size; study design; duration; physiological characteristics of participants; details of any intervention; background diet (including fiber content); fiber intervention; total fiber intake (background diet plus fiber intervention); measured outcome parameter; the method used to measure the outcome parameter; and a description of any adverse events. Extracted outcome data for total fecal wet weight (g/day) and transit time (h) included baseline and trial end values; change-from-baseline values; statistical significance of change values; and differences in the trial end value between the intervention and control arms.

### 2.3. Relevant Publications

Inclusion criteria were: (1) dietary fibers from a clearly-defined source; (2) healthy human subjects over 1 year of age; (3) measurement of regularity or related physiological functions associated with the intake of dietary fibers; (4) results of regularity or related physiological function described and discussed in the abstract; and (5) English-language publication.

Studies including mixed fibers and individuals with any pathological conditions (e.g., constipation, diarrhea, diverticular disease, ulcerative colitis and irritable bowel syndrome) were included in the database, but excluded from the analysis. Studies where dietary fibers were used in enteral formulas were excluded from the database.

#### 2.3.1. Grouping of Fibers by Source

Fibers were grouped by source of origin (*i.e.*, cereal, fruit or vegetable) and fermentability, which is on a continuum closely related to their chemical composition [7]. For the purpose of this review and these analyses, fibers were divided into the binary categories of more fermentable or less fermentable based on their predominant characteristics.

#### 2.3.2. Grouping of Fibers by Fermentability

Fermentability is often referred to as an important characteristic of dietary fiber in relation to health outcomes. However, no clear criteria are available to characterize levels of fermentability [15]. Therefore, we estimated the fermentability of all dietary fibers on the basis of information obtained in a non-systematic search on the combination of “fiber name” + “fermentable” or “fermentability”. The results were then discussed to determine eligibility (Prof. H.A. (Henk) Schols, Wageningen University & Research Centre, The Netherlands; personal communication). Supplemental Table S1 breaks out fibers according to fermentability. All fibers are fermentable to some degree, some more so than others. The binary categories of more fermentable and less fermentable are used in this report to enable quantitative analysis of the data.

### 2.4. Total Fecal Wet Weight and Transit Time

Included publications had a wide range of fiber types, study designs, methodologies and numbers of observations on fecal weight and transit time. Individual studies used a variety of methodologies to determine transit time, including indigestible dye, radio-opaque markers, polyethylene glycol and chromium sesquioxide. These approaches have the potential to yield different estimates of transfer time. To account for these differences and to provide the best possible estimates, we performed a weighted regression analysis on all available data categorized according to initial transit time with a cutoff point of 48 h. We also performed weighted linear regression analyses on fecal wet weight. Grouping by source of origin and fermentability enabled the generation of quantitative estimates on bowel function parameters. We calculated the slopes of regression coefficients and intercepts for changes in transit time based on initial times of <48 or ≥48 h.

### 2.5. Quality Ratings

We used two appraisal systems to assign study quality scores: (1) The Food Agency of Australia and New Zealand’s (FSANZ) criteria (0–15 points) [16]; and (2) Welch, *et al.*’s [17] criteria for human intervention studies (0–20 points).

### 2.6. Statistical Analysis

A meta-analysis according to PRISMA guidelines [14] was not feasible due to diverse study designs, the numbers of observations and methodologies. Instead, we used weighted regression analyses. We believe that this approach addresses the variability between studies and is best suited to an examination of the effects of cereal, fruit and vegetable fibers on fecal weight and transit time.

Data used for the control groups were based on the type of study. For placebo-controlled trials, we used data from the control arm. For uncontrolled trials, we used the baseline values of the study as the control. Some studies were dose-response trials. In these cases, the lowest dose was considered the control. In our search for and analysis of pertinent studies, we included all dietary fibers.

From available parameters on regularity, we reported total fecal wet weight and transit time. Dietary fibers were grouped according to food origin, estimated fermentability or a combination of food origin and estimated fermentability. We included studies in which there were 4 or more observations of any group of dietary fibers. In the analysis of the estimated fermentability of the dietary fibers, all observations were included, regardless of food origin. We generated means, standard deviations and 95% CIs for dietary fiber dose (g/day) and bowel function parameters.

We performed linear weighted regression analyses on fecal weight outcomes to develop quantitative estimates of the potential effect of dietary fibers on fecal weight. Regression analyses were weighted by the number of individuals in each study. Regression coefficients indicated the change in total fecal weight (g/g).

Observations in apparently healthy individuals suggest that whole gut transit time may vary between 40 and 60 h [18,19]. Based on the preponderance of evidence, we separated groups of individuals by transit time of <48 h (fast) or ≥48 h (delayed, slow). For the analysis of total transit time, we performed a multivariable weighted regression analysis that included initial transit time to account for known interactions. Regression coefficients indicate the change in transit time (h/g).

The *p*-values of the regression coefficients indicate a significant difference from zero. We also calculated the intercepts of the regression lines and their 95% confidence interval estimates. The *p*-values of the intercepts indicate a significant difference from zero. Regression analyses were not forced through zero. We performed a *t*-test to compare regression coefficients and intercepts. All analyses were conducted with SAS Version 9.4 (SAS, Cary, NC, USA). A *p*-value ≤ 0.05 was considered statistically significant.

## 3. Results

### 3.1. Study Characteristics

We searched PubMed and EMBASE once for intact cereal dietary fibers and a second time for other fibers. The first study identified was published in 1919 [20]. We reviewed studies through the end of 2013.

**Figure 1:**
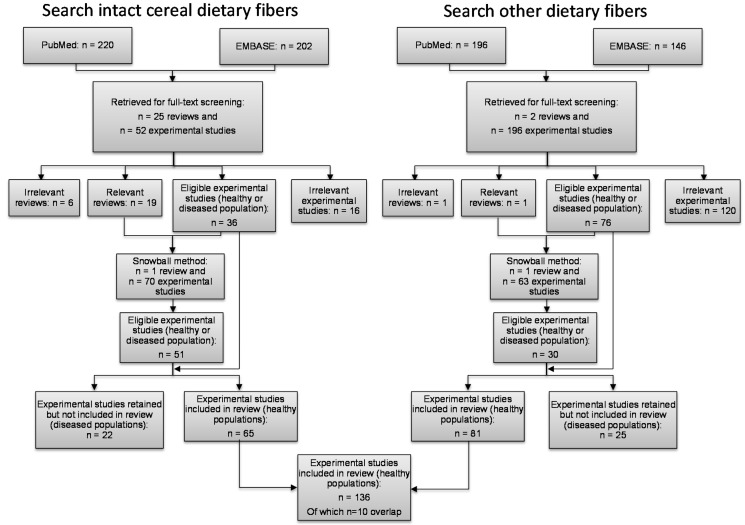
Search flow to identify and review publications. The figure shows the studies identified for inclusion in the review on the effects of dietary fiber on fecal weight and transit time. The overlap between the two searches indicates publications that tested dietary fibers from cereals and other sources.

Our initial search for cereal fibers identified a total of 422 publications (Figure 1). We retrieved and performed full-text screening on the 77 studies that met initial criteria for inclusion [2]. The snowball method identified 71 other studies. We excluded those with unhealthy populations, leaving 65 intervention trials for the analysis. Our second search for other fibers returned a total of 342 publications, of which 198 were retrieved for full-text screening. Of these, 76 were considered relevant. The snowball method identified 64 articles; of these, 30 were eligible for analysis. That left 81 studies after excluding those with unhealthy subjects.

Some publications reported on more than one type of dietary fiber. Therefore, our outcomes are based on the number of observations per parameter per dietary fiber. The analysis of total fecal weight and transit time in relation to food origin is based on 150 and 117 observations, respectively. The analysis of fermentability and total fecal wet weight and transit time is based on 206 and 122 observations.

The search included all eligible studies with all fibers (57 different fibers in total). The analyses included only dietary fibers from cereal, fruit or vegetable sources (a total of 26 different fibers).

### 3.2. Dietary Fibers

Cereal fibers studied included barley, corn germ, rice bran, rye bran, sorghum bran, corn bran, lignin, wheat bran, wheat starch, arabinoxylan, barley bran flour, oat bran, resistant starch and soluble corn fiber. The vegetable group included tomatoes, alfalfa, cabbage, carrot fiber, fructooligosaccharides (FOS), inulin and potatoes. Fruit fiber sources included oranges, kiwi fruit, apples, citrus pectin and prunes.

Table 1 shows comparable effects of dietary fibers from cereals and vegetables on total fecal wet weight and that non-fermentable dietary fibers contribute more to fecal weight than fermentable fibers (*p* < 0.05). Transit time ≥48 h is reduced by the same amount with fibers from cereals and vegetables, independent of fermentability.

### 3.3. Changes in Total Fecal Wet Weight

The majority (51%) of observations on total fecal wet weight came from cereal fibers. The slope of the regression coefficients for cereal dietary fibers was 2.17 (95% CI: 1.99–2.35; *p* < 0.0001) compared to 2.03 (95% CI: 1.59–2.46; *p* < 0.0001) for vegetable dietary fiber. The slope for fruit fibers was 0.88 (95% CI: 0.15–1.61; *p* = 0.02).

**Figure 2:**
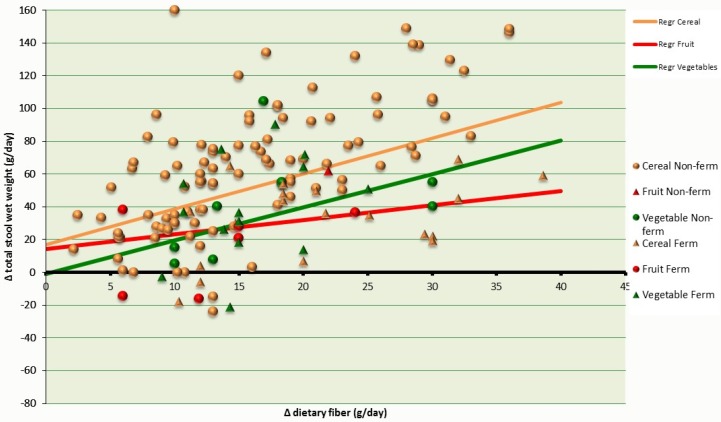
Changes in the weight of total fecal output (g/day) related to dietary fiber intervention (g/day) in healthy individuals. Dietary fiber is grouped according to food origin. Cereal Non-ferm: less fermentable dietary fibers from cereals; Fruit Non-ferm: less fermentable dietary fibers from fruits; Vegetable Non-ferm: less fermentable dietary fibers from vegetables; Cereal Ferm: more fermentable dietary fibers from cereals; Fruit Ferm: more fermentable dietary fibers from fruits; Vegetable Ferm: more fermentable dietary fibers.

The intercept of the regression line for cereal dietary fibers was 16.83 (95% CI: 13.41–20.23; *p* < 0.0001) compared to −0.88 (95% CI: −8.86–7.09; *p* = 0.83) for vegetable fibers. The intercept for fruit fiber was 14.27 (95% CI: 5.03–23.50; *p* = 0.00). Figure 2 shows the effect of the interventions with dietary fiber (g/day) on total fecal wet weight in healthy individuals, with dietary fibers grouped according to food origin.

### 3.4. Changes in Transit Time

The change in transit time for cereal fibers with an initial time <48 h was 0.20 (95% CI: 0.01–0.37; *p* = 0.03); for ≥48 h, it was 0.50 (95% CI: −0.61 to −0.39; *p* < 0.0001). The intercept of the regression line for initial transit time <48 h was −8.95 (95% CI: −12.36 to −5.54; *p* < 0.0001); for ≥48 h, it was −10.23 (95% CI: −12.18 to −8.26; *p* < 0.001).

Too few observations (<5) were available for a valid regression for fruit dietary fibers with an initial transit time <48 h. Only one observation was available for vegetable dietary fiber with a transit time <48 h.

### 3.5. Changes in Total Fecal Wet Weight by Fermentability

**Figure 3:**
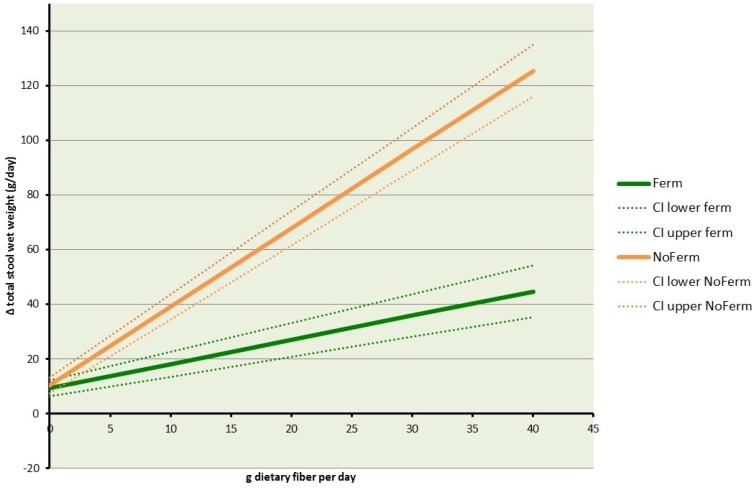
Changes in total fecal wet weight (g/g dietary fiber) and corresponding confidence intervals organized according to fermentability. Ferm: more fermentable; NoFerm: less fermentable; CI: confidence interval.

The slope of the regression coefficients for fermentable fibers was 0.89 (95% CI: 0.72–1.04; *p* < 0.0001). For non-fermentable fibers, it was 2.88 (95% CI: 2.71–3.04; *p* < 0.0001). The respective intercepts were 9.24 (95% CI: 6.24–12.24; *p* < 0.0001) and 10.34 (95% CI: 7.37–13.29; *p* < 0.0001). Figure 3 shows the changes in total stool wet weight (g/g dietary fiber) and their confidence intervals according to fermentability.

### 3.6. Changes in Transit Time by Fermentability

For fermentable dietary fibers, the slope of the regression coefficients for change in transit time with an initial time <48 h was 0.15 (95% CI: 0.03–0.26; *p* = 0.01). For initial transit time ≥48 h, it was −0.48, *p* < 0.0001. The respective intercepts were −0.43 (95% CI: −2.85–1.98; *p* = 0.72) and 7.23 (95% CI: 4.55–9.89; *p* < 0.0001).

The slope of the regression coefficients for non-fermentable fibers with an initial transit time <48 h was 0.14 (95% CI: −0.03–0.30; *p* = 0.11). For initial transit time ≥48 h, it was −0.45 (95% CI: −0.03–0.30; *p* < 0.0001). The respective intercepts were −7.69 (95% CI: −10.962 to −4.425; *p* < 0.0001) and −12.13 (95% CI: −13.751 to −10.518; *p* < 0.0001).

## 4. Discussion

Outcomes from this study show that estimated fermentability determines the role of fiber in total fecal wet weight. Less fermentable dietary fibers from cereals, such as wheat bran, contribute most to total fecal wet weight. This is likely due to their higher water binding capacity compared to more fermentable dietary fibers, as well as greater resistance to fermentation from colonic bacteria [2,21].

Observations in healthy individuals suggest that a normal physiological transit time varies between 40 and 60 h [18,19]. In general, transit time is normalized by increasing dietary fiber, regardless of the fiber type. Our findings are consistent with those of de Vries, *et al.* [2]. When transit time is already optimal, *i.e.*, between 24 and 48 h, additional dietary fiber does not appear to alter it.

The U.S. Dietary Reference Intakes [22] recommend a daily dietary fiber intake of 14 g/1000 kcal or 25 g/day for women and 38 g/day for men. Eating a variety of cereals, fruits and vegetables can promote regularity [23]. Increasing evidence suggests that the health benefits of fruits, vegetables, grains and other plant foods can be attributed to the synergy or interactions of bioactive compounds and other nutrients in whole foods [24], some of which are considered “co-passengers” of dietary fibers [25]. Dietary guidelines from Europe and the USA are consistent in recommending that people eat a variety of fibers from different food sources to achieve a healthy diet [11].

Consumer research suggests that many Americans believe that they are getting enough daily fiber, but there is confusion around which foods provide it and how much fiber is needed for good health [26]. As a result, the public consumes approximately half the recommended amount of dietary fiber per day [27,28]. Adequate intakes of fiber are associated with reduced risk for cardiovascular disease, cancer, diabetes, certain gastrointestinal disorders and obesity [27].

Most research indicates that regularity is a hallmark of healthy bowel function [7]. Studies indicate that fibers from fruits and vegetables produce some of the same effects as the addition of cereals to the diet, promoting regularity through significantly increased fecal weight and decreased fecal transit time [29]. Of all of the fibers, the composition of those from fruit tend to be more fermentable and soluble than those from vegetables or cereals, which are likely to be the least fermentable and, therefore, have the greatest impact on fecal wet weight and transit time.

Since the review on fiber and bowel function by Cummings [1], much research has been done on the effects of dietary fiber on fecal weight and transit time associated with the intake of individual dietary fibers. The strengths of this study include the large number of publications evaluated (*n* = 93) and its comprehensiveness, *i.e.*, an analysis based on all major food sources of dietary fiber (cereals, fruits and vegetables). We covered 26 different types of fibers categorized into three food-based sources, with clinical trials dating back to 1919 [20].

This report has some limitations. The overall quality of numerous early studies did not meet current clinical trial standards [16,17]. This might have caused confounding we could not control for. However, scientific approaches develop over time. The designs and methods of older reports may differ from the most recent interventions, yet their outcomes have value.

In general, older studies had smaller sample sizes than more current research. To adjust for this potential confounding, we used weighted regression analyses with the assigned weight determined by the number of study subjects. FSANZ scores ranged from 0 to 15 points [16], and Welch, *et al.* scores were between 6 and 18 [17], with lower quality most often due to poor descriptions of subjects, recruitment and methodology.

Intervention designs varied widely, from single arm pilot studies to randomized, double-blind, placebo-controlled trials. Participants per study ranged from 4 to 81 and duration from 5 days to 14 weeks. The number of studies for vegetable and fruit fibers was scant, and there was substantial individual variability in measured parameters. Single arm reports required the use of the starting value as the control. These factors precluded quality assessment and a meta-analysis according to PRISMA criteria.

To develop statistical estimates, it was necessary to categorize the fibers into three heterogeneous groups. This might have reduced the comparability of the outcomes. Furthermore, many weighted regression intercepts differed from zero, suggesting confounding. Despite these limitations, our findings corroborate the body of evidence showing that increased fiber from all sources normalizes transit time, increases fecal weight and improves regularity [1,30].

A worldwide increase in the incidence of gastrointestinal disorders has created an immediate need to identify safe and effective interventions to maintain regularity [31,32]. In the U.S. alone, nearly 5% of the population suffers from functional constipation [33]. Increasing dietary fiber intake to recommended levels could save in excess of $12.7 billion a year in direct medical costs to treat this condition alone [33].

Despite possible confounding, this study shows that the less fermentable a fiber is, the more it contributes to total fecal wet weight. It also indicates that slow transit time (≥48 h) can be normalized by increasing dietary fiber, regardless of the fiber type consumed. These findings suggest that adequate intake of cereal, fruit and vegetable fibers from a variety of food sources contribute to healthy eating habits and have the potential to increase population-wide levels of regularity and healthy bowel function, thereby reducing the economic burden imposed by gastrointestinal disorders.

## Figures and Tables

**Table 1:** Summary of regression analyses of changes in total fecal wet weight and transit time according to food source and fermentability ^1,2,3^.

Fecal Weight & Transit Time	Fermentability	Cereal	Fruit	Vegetable	All Dietary Fiber
Total Fecal Wet Weight (g/g DF)	More Fermentable	**1.29 (1.02; 1.56) ^b,y^**	**0.28 (−0.50; 1.06) ^c^**	**2.91 (1.96; 3.86) ^a^**	**0.89 (0.73; 1.05)**
		*0.51 (−5.92; 6.95)* ^a^	*18.59 (9.32; 27.86)* ^a^	*−10.56 (−26.6; −5.56)* ^c^	*9.24 (6.24; 12.24)*
		(*n* = 21)	(*n* = 6)	(*n* = 14)	(*n* = 70)
	Less Fermentable	**3.39 (3.22; 3.56) ^b,x^**	-	**1.91 (1.63; 2.20) ^b^**	**2.88 (2.72; 3.04)**
		*6.22 (3.26; 9.18)* ^a^		−*6.02 (−11.94; −0.11)* ^b^	*10.34 (7.38; 13.30)*
		(*n* = 100)	(*n* = 1)	(*n* = 8)	(*n* = 136)
	More and Less Fermentable	**2.17 (2.00; 2.35) ^a^**	**0.88 (0.16; 1.61) ^b^**	**2.03 (1.6; 2.46) ^a^**	
		*16.88 (13.42; 20.24)* ^a^	*14.27 (5.04; 23.50)* ^a^	*−0.88 (−8.86; 7.10)* ^b^	
		(*n* = 121)	(*n* = 7)	(*n* = 22)	
Transit Time Initial <48 h (h/g DF)	More Fermentable	**0.19 (0.01; 0.38)**	-	-	**0.15 (0.04; 0.27) ^x^**
		*1.90 (−3.04; 6.83)* ^x^			*−0.43 (−2.85; 1.98)* ^a,x^
		(*n* = 4)	(*n* = 2)	(*n* = 1)	(*n* = 8)
	Less fermentable	**−0.05 (−0.22; 0.11)**	-	-	**0.14 (−0.03; 0.31) ^x^**
		*−6.88 (−9.80; −3.95)* ^y^			−7.69 (−10.96; −4.43) ^b,x^
		(*n* = 16)	(*n* = 0)	(*n* = 0)	(*n* = 16)
	More and Less Fermentable	**0.20 (0.02; 0.37) ^x^**	-	-	
		*−8.95 (−12.37; −5.54)*			
		(*n* = 20)	(*n* = 2)	(*n* = 1)	
Transit Time Initial ≥48 h (h/g DF)	More Fermentable	**−0.69 (−0.92; −0.47)**	-	**−0.87 (−1.61; −0.14)**	**−0.48 (−0.62; −0.33) ^y^**
		*16.98 (12.57; 21.39)* ^x^		*9.31 (−2.00; 20.62)*	*7.23 (4.56; 9.90)* ^y^
		(*n* = 9)	(*n* = 2)	(*n* = 8)	(*n* = 24)
	Less Fermentable	**−0.67 (−0.76; −0.58)**	-	**−0.47 (−0.66; −0.27)**	**−0.45 (−0.54; −0.36) ^y^**
		*−10.82 (−12.40; 9.23)* ^y^		*−1.75 (−5.51; 2.01)*	*−12.13 (−13.75; −10.52)* ^y^
		(*n* = 63)	(*n* = 1)	(*n* = 9)	(*n* = 74)
	More and Less Fermentable	**−0.50 (−0.61; 0.39) ^a,y^**	-	**−0.52 (−0.69; −0.35) ^a,b^**	
		*−10.23 (−12.19; −8.27)* ^c^		*0.46 (−2.72; 3.64)* ^b^	
		(*n* = 72)	(*n* = 3)	(*n* = 17)	

^1^ Bold figures represent the slope of the regression line; italic figures represent the intercept of the regression line; ^2^ the results of the table are based on all available data on the effects of dietary fibers on total stool weight and transit time, including the results of dietary fiber from other food sources; ^3^ regression coefficients and intercepts have been statistically compared. Comparisons within rows are presented with ^a^, ^b^ and ^c^. Comparisons within columns are presented with ^x^ and ^y^. Any two items with no letter in common are significantly different, with a *p* < 0.05. ^4^ Abbreviation: DF, dietary fiber.

## Supplementary Materials

The following is available online at http://www.mdpi.com/2072-6643/8/3/130/s1, Table S1: Table with estimates of the fermentability of dietary fibers.
