# Supplementary material for: Effects of Cereal, Fruit and Vegetable Fibers on Human Fecal Weight and Transit Time: A Comprehensive Review of Intervention Trials
Source: Nutrients. 2016 Mar 2;8(3):130. doi: 10.3390/nu8030130 (PMC4808860; doi:10.3390/nu8030130)
Supplement: Supplementary file 1 [file nutrients-08-00130-s001.docx]

**Supplementary Materials: Effects of Cereal, Fruit
and Vegetable Fibers on Human Fecal Weight and Transit Time: A Comprehensive Review of Intervention Trials**

# Jan de Vries, Anne Birkett, Toine Hulshof, Kristin Verbeke and Kernon Gibes

**Table S1.** Table of dietary fibers used in the intervention studies included in the analysis, including information on food origin and estimated fermentability.

| **Type of Dietary Fiber** | **Food Origin** | **Fermentability** |
| --- | --- | --- |
| Levan | bacterial | less fermentable |
| Xylan | bacterial | more fermentable |
| Barley | cereal | less fermentable |
| Corn germ | cereal | less fermentable |
| Rice bran | cereal | less fermentable |
| Rye bran | cereal | less fermentable |
| Sorghum bran | cereal | less fermentable |
| Corn bran | cereal | less fermentable |
| Lignin | cereal | less fermentable |
| Wheat bran | cereal | less fermentable |
| Wheat starch | cereal | less fermentable |
| Arabinoxylan | cereal | more fermentable |
| Barley bran flour | cereal | more fermentable |
| Oat bran | cereal | more fermentable |
| Resistant starch | cereal | more fermentable |
| Soluble Corn Fiber | cereal | more fermentable |
| Oranges | fruit | less fermentable |
| Kiwi fruit | fruit | less fermentable |
| Apple fiber | fruit | more fermentable |
| Citrus pectin | Fruit | more fermentable |
| Prunes | fruit | more fermentable |
| Pullulan | molds | more fermentable |
| Cotton seed hull | other | less fermentable |
| Fibrotein | other | less fermentable |
| Carboxymethylcellulose | other | less fermentable |
| Cellulose | other | less fermentable |
| Cocoa (roasted) | other | less fermentable |
| Cocoa bran | other | less fermentable |
| Flax seed | other | less fermentable |
| Non Starch Polysaccharide | other | less fermentable |
| Psyllium | other | less fermentable |
| Arabinogalactan | other | more fermentable |
| GOS | other | more fermentable |
| Maltitol | other | more fermentable |
| Xanthan gum | other | more fermentable |
| Ispaghula husk | plant | less fermentable |
| Karaya gum | plant | less fermentable |
| Locust bean gum | plant | less fermentable |
| Sugar beet | plant | less fermentable |

# Table S1. *Cont.*

| **Type of Dietary Fiber** | **Food Origin** | **Fermentability** |
| --- | --- | --- |
| Glucomannan | plant | more fermentable |
| Guar gum | plant | more fermentable |
| Konjac powder | plant | more fermentable |
| Peas | pulses | less fermentable |
| Soy oligosaccharide | pulses | more fermentable |
| Soy polysaccharide | pulses | more fermentable |
| Agar agar | sea plants | less fermentable |
| Methylcellulose | synthesized | less fermentable |
| Polydextrose | synthesized | more fermentable |
| Soluble fiber dextrin | synthesized | more fermentable |
| Tomatoes | vegetable | less fermentable |
| Alfalfa | vegetable | less fermentable |
| Cabbage | vegetable | less fermentable |
| Carrot fiber | vegetable | more fermentable |
| FOS | vegetable | more fermentable |
| Inulin | vegetable | more fermentable |
